# Diagnosis and Surgical Management of a Paratesticular Cyst in a Rehabilitating Juvenile Male Green Turtle (*Chelonia mydas*)

**DOI:** 10.3389/fvets.2020.00069

**Published:** 2020-02-27

**Authors:** Tatiana C. Weisbrod, Nicole I. Stacy, Nancy L. Stedman, Terry M. Norton

**Affiliations:** ^1^Department of Comparative, Diagnostic, and Population Medicine, College of Veterinary Medicine, University of Florida, Gainesville, FL, United States; ^2^Busch Gardens Tampa, Tampa, FL, United States; ^3^Georgia Sea Turtle Center/Jekyll Island Authority, Jekyll Island, GA, United States

**Keywords:** anemia, developmental abnormality, epididymal cyst, Mullerian duct, surgery

## Abstract

A juvenile green turtle (*Chelonia mydas*) undergoing rehabilitation for cold stunning exhibited an asymmetric bulging of the left caudal plastron and was diagnosed with a large intra-coelomic mass based on radiographical findings. Ultrasonography further identified a fluid-filled structure within the caudal coelom. Cytological evaluation of fluid obtained from the structure was consistent with a transudate, and thus, a cyst of unknown origin was suspected. Computed tomography imaging was pursued to further characterize the extent and location of the mass, which occupied ~50% of the total coelomic cavity volume. Conservative management with monitoring and occasional drainage of the mass did not result in improvements; thus, an exploratory laparoscopy for further investigation and surgical planning was elected. Intra-coelomic surgery was performed to remove a thick-walled cystic mass associated with the left gonad. Histopathology confirmed a paratesticular cyst continuous with, and possibly originating from, the epididymis. Post-surgical recurrence of the cyst was not appreciated, and the animal was successfully released 1 year after admission. Unrelated to the cyst, the turtle developed acute severe anemia on two occasions throughout rehabilitation that responded to modification of antimicrobial treatment and subsequent steroid administration. To the authors' knowledge, this is the first report of a paratesticular cyst in a reptile.

## Introduction

A free-ranging juvenile green turtle (*Chelonia mydas*) of unknown sex, weighing 2.4 kg was admitted to the Georgia Sea Turtle Center on Jekyll Island, Georgia, for rehabilitation after cold stunning at Fernandina Beach, Florida, in February 2016. The turtle was found to be thin (body condition score 2/5) with mild algae and epibiota coverage on the carapace. Hematological and plasma biochemical analyses were consistent with typical findings in cold-stunned green turtles (1), including heterophilia consistent with systemic inflammation; hypoglycemia, hypoproteinemia, hypoalbuminemia, and hypocalcemia likely secondary to emaciation and anorexia; elevated uric acid presumably from dehydration or possibly renal disease; and elevated creatine kinase from muscle injury or exertion (Table 1). Packed cell volume (PCV) was normal at this time. Initial whole-body radiographs were unremarkable. Medical treatment was initiated with ceftazidime (20 mg/kg BW, every 3 days for 39 days, subcutaneously), lactated Ringer's solution (LRS) with 5% dextrose (5 ml/kg BW, as indicated by daily bloodwork monitoring, intravenously), and 23% calcium gluconate (0.2 ml/kg BW, once daily until normalized, subcutaneously).

**Table 1:** Select hemogram and plasma biochemical findings over time in a cold-stunned green turtle (*Chelonia mydas*).

**Days from admission**	**0**	**56**	**81**	**98**	**270**	**297**	**345**	**Reported data**
**Clinical time point**	**Admit**	**First anemic** ** event**	**Diagnosis of** ** mass**	**Second anemic** ** event**	**Pre-** **operative**	**2 weeks post-** **operative**	**Pre-** **release**	
Creatine kinase, μmol/L	80.9	18.5	6.1	10	7.7	54.9	20.4	NR
Albumin, g/L	5	12	16	14	12	8	8	6–21
Total protein, g/L	17	33	50	41	31	23	25	26–69
Globulin, g/L	12	21	34	27	19	15	17	19–52
Uric acid, μmol/L	273.6	47.6	83.3	77.3	47.6	53.5	41.6	29.7–208.2
Glucose, mmol/L	1.4	6.1	12.4	6.9	5.4	5.4	5.8	4.8–9.3
Calcium, mmol/L	1	1.3	1.7	1.5	1.2	1.2	1.1	0.4–3
Phosphorus, mmol/L	2.4	3.2	2.8	3.1	3.3	3.2	2.3	1.2–3.5
Potassium, mmol/L	4.6	4.1	3.7	4.8	4.6	3.6	4.7	4.1–6.9
Chloride, mmol/L	121	124	106	130	115	124	116	100–130
Sodium, mmol/L	155	158	158	157	155	156	156	157–183
PCV L/L	0.28	0.10/0.11	0.18	0.13	0.31	0.12	0.32	0.17–0.38
TS g/L	16	36	51	44	34	20	26	NR
Immature RBC/100 (PCV <25)	NR	32	84	76	NR	22	NR	NR
WBC estimate × 10^9^/L	24.50	9.30	34.50	33.00	14.10	89.00	10.20	1.76–22.40
Absolute heterophils × 10^9^/L	18.00	3.50	19.00	20.00	7.50	71.00	6.20	0.23–2.59
Absolute immature heterophils × 10^9^/L	0.00	0.00	0.69	2.00	0.00	0.89	0.00	NR
Absolute lymphocytes × 10^9^/L	4.90	3.90	10.00	7.30	4.50	12.00	2.10	3.42^*^
Absolute monocytes × 10^9^/L	1.70	1.70	4.80	3.30	1.40	2.70	0.51	0.18–2.92
Absolute eosinophils × 10^9^/L	0.25	0.09	0.00	0.66	0.42	1.80	0.92	0.37–5.16
Absolute basophils × 10^9^/L	0.00	0.19	0.00	0.00	0.28	0.00	0.51	0–0.13
Thrombocytes	Adequate	Adequate	Adequate	Adequate	Adequate	Adequate	Adequate	NA
Erythrocyte morphology	Occ. fragments	Moderate anisocytosis and polychromasia	Moderate anisocytosis and polychromasia	Moderate anisocytosis and polychromasia	NSCF	Moderate anisocytosis and polychromasia	NSCF	NA
WBC morphology	NSCF	NSCF	Mild left-shift, 1+ toxicity	Moderate left-shift, 1+ toxicity	NSCF	Mild left shift	NSCF	NA

Plasma biochemical reference ranges are minimum and maximum values from 100 free-ranging juvenile green turtles (2). Hemogram reference ranges are minimum and maximum values (unless otherwise noted) from 23 juvenile and 5 mature free-ranging green turtles (3); NR, not reported; NA, not available; NSCF, no significant cytological findings;

* *number represents the mean*.

Bloodwork, appetite, and weight steadily improved over the first 4 weeks after admission; however, 8 weeks from initial presentation, the turtle developed an acute, regenerative, non-hemolytic anemia (Table 1). A blood culture was negative, ceftazidime administration was discontinued, and broad-spectrum antimicrobial therapy was instituted with amikacin (5 mg/kg BW first dose followed by 3 mg/kg BW, every 3 days for 46 days, subcutaneously) and ampicillin (30 mg/kg BW, once daily for 17 days, subcutaneously) followed by amoxicillin/clavulanic acid (30 mg/kg BW, once daily for 29 days, orally), and dexamethasone sodium phosphate at an immunosuppressive dose (0.5 mg/kg BW, once daily for 10 days, then every other day for 6 days). The turtle developed hyperglycemia with concurrent steroid administration, which normalized upon its discontinuation, and PCV improved over 3 weeks.

**Figure 1:**
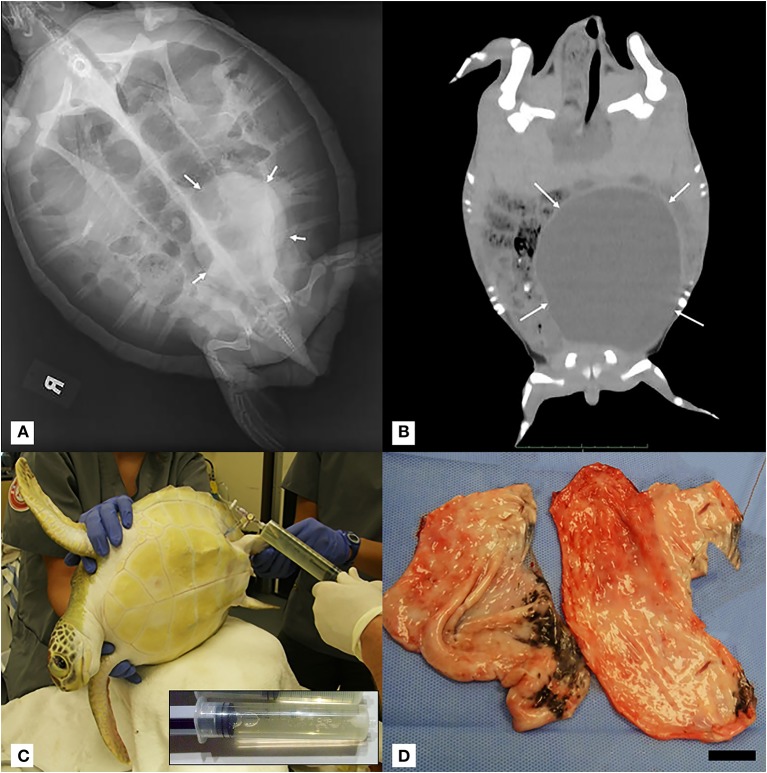
**(A)** Dorsoventral projection full-body radiograph of a juvenile green turtle (*Chelonia mydas*) showing a large rounded well-demarcated mass with soft tissue opacity within the left caudal coelom (white arrows). **(B)** Coronal mid-coelomic CT section of a green turtle (*Chelonia mydas*) showing a large rounded soft tissue/fluid opacity mass (white arrows) within the left caudal coelom displacing gastrointestinal contents dorsally and to the right. CT was performed 2 weeks after initial radiographic diagnosis of mass, and no fluid was aspirated during this interval. Progression of gross left-sided plastron distention was noted. **(C)** Positioning of turtle in right lateral recumbency for left pre-femoral fine-needle fluid aspiration of intra-coelomic cyst. Inset: Representative 60-ml syringe filled with transudate fluid from cyst aspiration. **(D)** Gross image of cyst in two sections post-surgical removal. Scale bar = 2.5 cm.

During routine physical examinations, about 1 month following the development of the acute anemia, the left side of the plastron was noted to bulge asymmetrically compared to the right side. Whole-body radiographs identified a large round soft tissue mass within the caudal left coelom that was not appreciated on prior images (Figure 1A). Follow-up CT imaging confirmed the presence of a large mass comprising ~50% of the total coelomic volume within the left caudal coelom (Figure 1B). Differentials included cyst, abscess, hematoma, neoplasia (e.g., fibropapillomatosis), or much less likely an anatomical abnormality. Ultrasound of the region from the left pre-femoral window showed multiple anechoic structures with cystic appearance. An ultrasound-guided fine-needle aspirate of the fluid-filled structure yielded a clear pale-yellow, low protein (total solids 4 g/dl), acellular (white blood cell estimate 0/μl) fluid most consistent with a transudate (Figure 1C). Coinciding with the administration of steroids and the diagnosis of the mass were marked heterophilia, lymphocytosis, and monocytosis with left shift and mild toxic change (Table 1), consistent with chronic-active inflammation, which improved slowly over 4 weeks. The turtle developed anemia a second time about 6 weeks after the first (Table 1), and a second blood culture was obtained and was negative. This event resolved without medical intervention over 3 weeks.

Repeat fluid aspirations from the mass were performed on a near-weekly interval for both therapeutic and diagnostic monitoring purposes. The fluid was similar in consistency and color to the initially diagnosed transudate, and when successful, an average of 100 ml was removed during each aspiration attempt. One of the samples appeared cloudy and cytologically disclosed marked histiocytic and heterophilic inflammation with the presence of extracellular and phagocytized bacteria. It is unclear whether this sample represented fluid from the coelomic mass or coelomic effusion. Accidental intestinal aspiration was not considered a possibility given the location of fluid aspiration. Based on these findings, antimicrobial administration with metronidazole (20 mg/kg BW, once daily for 40 days, orally) and enrofloxacin (5 mg/kg BW, once daily for 40 days, orally) was initiated for coverage against enteric pathogens. Next, further diagnostics to delineate the origin of the mass were pursued.

The turtle was anesthetized for exploratory laparoscopy with dexmedetomidine (74 μg/kg BW, intravenously), butorphanol (0.45 mg/kg BW, intravenously), and alfaxalone (0.5 mg/kg BW, intravenously). The patient was intubated (4 mm uncuffed endotracheal tube) and maintained with sevoflurane (variable from 0.5 to 3%). A 2-cm, left pre-femoral skin incision was made, and an examination trocar/cannula was inserted followed by a 2.7-mm 30° video laparoscope. A single large mass was visualized within the caudal coelom as well as three to four cystic structures in the region of the left gonad. These were initially interpreted as ovarian tissue; however, it was later determined histologically that the turtle was male. It was impossible to determine the origin of the mass by laparoscopy, and no free coelomic fluid was appreciated. The surgical site was closed with 4-0 polydioxanone (PDSII, Ethicon, Sommerville, NJ, USA) using a horizontal mattress suture pattern. The dexmedetomidine was reversed with atipamezole (0.74 mg/kg BW, intravenously; then 0.22 mg/kg BW, intravenously 2 h later due to slow recovery), and the turtle recovered uneventfully. Two weeks later, the turtle was briefly sedated with dexmedetomidine (50 μg/kg BW, intravenously) for cloacoscopy and contrast cystogram. Iodinated contrast media (20 ml, Iohexol) was instilled into the cloaca through the rigid endoscope. Results of this examination showed normal cloacal, ureteral, and urinary bladder anatomy without evidence of an associated mass.

**Figure 2:**
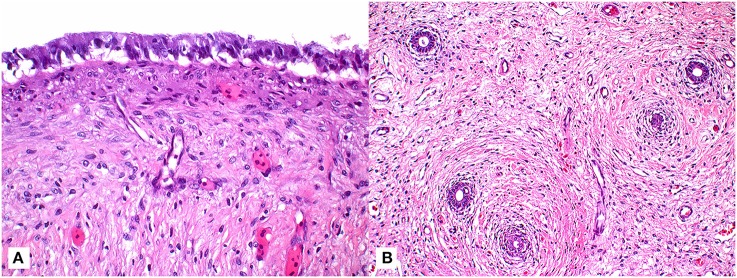
Tissue sections of a paratesticular cyst surgically removed from a male green turtle (*Chelonia mydas*). **(A)** Photomicrograph of tissue section demonstrating columnar epithelial cells overlying maturing fibroplasia of cyst wall (63x; hematoxylin and eosin stain). **(B)** Lower magnification photomicrograph highlighting the presence of ducts (arrows) entrapped within the connective tissue of cyst wall (20x; hematoxylin and eosin stain).

Given the persistence of the mass and the need for repeated fluid aspirations, surgical removal was performed ~6 months after the initial discovery. Pre-operative bloodwork revealed a mildly low calcium (1.2 mmol/L) (Table 1) so a single dose of calcium gluconate (calcium gluconate 23%, 0.2 ml/kg BW, subcutaneously) was administered. The turtle was anesthetized with dexmedetomidine (75 μg/kg BW, intravenously), ketamine (1 mg/kg BW, intravenously), and butorphanol (0.4 mg/kg BW, intravenously), received 30 ml LRS IV at the time of induction, and a local anesthetic block was performed on the left pre-femoral surgical site (0.5 ml lidocaine, 2% solution, in 0.5 ml sodium bicarbonate). The turtle was intubated with a 5-mm uncuffed endotracheal tube, maintained on inhalant sevoflurane (0.5–1%), and manually ventilated at a rate of two breaths per minute. A surgical approach was made to the left pre-femoral region with the turtle in right lateral recumbency. A combination of laparoscopic and standard surgical instrumentation was used to visualize and completely excise the mass. The structure appeared to be in close association with the left kidney and gonad, but a definitive origin could not be determined. Heavy bleeding occurred during the procedure, and hemostasis was achieved via direct ligation of blood vessels. Sectioning of the mass into two portions facilitated its removal, and the coelomic cavity was thoroughly flushed with normal saline and suctioned prior to closure (Figure 1D). The incision was closed in two layers with 4-0 PDS in a simple interrupted pattern. Dexmedetomidine was reversed with atipamezole (75 mg/kg BW, intravenously), and the turtle recovered slowly, but smoothly. The turtle received tramadol (5 mg/kg BW, every other day for two doses, subcutaneously) and meloxicam (0.5 mg/kg BW, once daily for 5 days, subcutaneously) post-operatively. Bloodwork performed post-operatively showed an increase in creatine kinase activity, anemia, hypoproteinemia, and an extreme leukocytosis characterized by heterophilia, lymphocytosis, monocytosis, and eosinophilia (Table 1), consistent with post-surgical chronic-active inflammation, blood loss, and muscle injury. The severe anemia with concurrent hypoproteinemia was likely secondary to blood loss and/or systemic inflammation post-surgery. Histopathological evaluation of the cystic mass confirmed a 3- to 4-mm-thick wall of vascular dense collagenous connective tissue rarely covered by necrotic and sloughing cuboidal to columnar epithelium arranged in a single layer. The connective tissue was continuous with the stroma of the immature epididymis but did not involve the testis. Rarely, the connective tissue entrapped small ducts lined by a single layer of cuboidal epithelial cells with occasional hyperplasia (Figure 2). Connective tissue was arranged concentrically around some entrapped ducts. Ducts were morphologically similar to those of the immature epididymis. Cilia were not identified on any epithelial cells of the cystic mass. The cyst wall and adjacent testicular and epididymal tissue were stained immunohistochemically for smooth muscle actin using a commercially available mouse monoclonal antibody and an automated staining platform following the manufacturer's recommendations (clone 1A4; Roche Diagnostics, Indianapolis, IN, USA). Vascular smooth muscle in these tissues exhibited strong cytoplasmic immunoreactivity, but positive reactivity was not detected elsewhere in the tissues. A granuloma surrounding remnants of a degenerate metazoan parasite was also present within the connective tissue.

Following surgery, the turtle received supportive care including iron dextran supplementation (5 mg/kg BW, subcutaneously), and antimicrobial administration with amoxicillin/clavulanic acid (30 mg/kg BW, once daily for 30 days, orally) and amikacin (3 mg/kg BW, every 3 days for 36 days, subcutaneously) was continued. Over 2 months, the patient improved steadily, and the anemia fully resolved. Physical examination and recheck radiographs revealed that the cyst had not regrown 2 months following the surgery. Pre-release bloodwork was normal, and the turtle was released near his original stranding location off the coast of Anastasia State Park, St. Augustine, Florida, about 1 year (i.e., 348 days) after admission.

## Discussion

This is the first report of a cyst of reproductive origin in a reptile species, specifically a marine turtle, which represented an unusual clinical challenge given the unprecedented finding and the extent of the intra-coelomic extension of the mass. Furthermore, we describe the medical management of acute anemia in this green turtle patient.

Reproductive cysts are infrequently reported in the veterinary literature, and information is limited to case reports in mammalian species. Reports of intra-coelomic cysts in reptiles are rare and limited to non-reproductive renal cysts. In mammalian species, epididymal cysts and testicular cysts have been reported most frequently and were largely incidental findings as they are rarely associated with reproductive disorders (4–9). Gartner duct (Wolffian duct) cysts have been reported in female dogs and have been associated with urinary dysfunction and/or additional congenital anomalies (10, 11). This latter condition occurs due to failure of Wolffian duct regression in females, and an analogous condition can occur in males. Mullerian duct cysts can result from failure of this embryological structure to fully regress in males. In humans, Mullerian duct cysts are relatively rare occurring in only 1–5% of the young, adult male population and are not typically associated with additional urogenital abnormalities (12). Cysts are typically benign and compared to other described cyst types, which arise from adjacent reproductive structures, can be relatively large, fluid-filled, and non-communicating with other organs (13, 14). Mullerian duct formation and regression in reptiles, including chelonians, seem to occur in a fashion analogous to mammals with full regression seen by hatching in red-eared sliders (*Trachemys scripta elegans*) (15, 16). In marine turtles, however, Mullerian ducts are recognized to occasionally persist as non-functioning tubular structures adjacent to the testes or kidneys (17). The anatomical location of the cyst in this case could be consistent with either an epididymal cyst or a Mullerian duct cyst, although the latter may be less likely based upon the absence of smooth muscle and presence of epididymal-like ducts in the cyst wall. A congenital, rather than acquired, cyst is considered to be most likely based on the age of the animal, histological appearance, and lack of evidence for prior trauma. Cyst detection due to enlargement was coincident with convalescence, and it is presumed that correction of initial dehydration and poor nutritional plane, in addition to return to normal behaviors, resulted in continuous fluid accumulation within the cyst. Although such cysts are considered incidental findings in many species, the size, extent, and continued enlargement of the cyst in this case was concerning for potential complications. In humans, urinary retention, recurrent infections, urogenital inflammation, and reduced fertility have been associated with various cyst types (14). Surgical removal of the entire cyst is typically curative as was considered to be in this case (18). In marine turtles, the testes are attached to the kidneys via the mesorchium and lie in close proximity to renal arteries and veins (17). This anatomical relationship makes urogenital surgical intervention challenging as there is a high risk of marked blood loss. Blood loss was significant during surgery in this case; however, meticulous dissection and prompt hemostasis helped reduce the risk of potentially fatal hemorrhage. Owing to close association with the cyst, a small section of immature epididymis was removed. The potential impact of partial, unilateral epididymectomy on reproductive capacity in reptiles is unknown, and changes in testicular architecture, spermatogenesis, or ejaculate sperm concentration are possible (19). Normal functioning and fertility may be maintained provided that the contralateral reproductive tract remains unaffected (20). Long-term follow-up was not possible as this turtle was released, but cyst recurrence was absent several months prior to release, and the PCV had normalized (Table 1).

Unrelated to the diagnosis and management of the cyst were the two episodes of anemia in this animal. Anemia of varying severity can be observed in cold-stunned marine turtles of any species admitted to rehabilitation and typically becomes more apparent upon rehydration (1). The acute severe regenerative anemia that occurred 2 months into rehabilitation in this patient, however, was not a typical finding for cold-stunned marine turtles. Several authors have identified cases of acute hemolytic anemia at facilities with convalescing marine turtles, although no clear cause has been identified to date (TN, NS). Light gray/greenish plasma indicative of biliverdinemia was reported for blood samples collected during anemic events of the current patient, presumptively suggesting extravascular hemolysis resulting in the release of biliverdin from heme degradation (21). Possible causes for acute hemolytic anemia seen in cold-stunned marine turtles include underlying septicemia, adverse drug reaction, and/or an association with cold stunning (e.g., increased osmotic fragility or other unknown causes). Two blood cultures obtained during anemic events in this case were negative; however, given antimicrobial administration concurrent with sampling as well as the likely differing susceptibility of certain bacterial species at varying incubation temperatures, this does not exclude septicemia as a possible underlying cause (22). Leukogram findings also supported systemic inflammation for which considerations include non-infectious and infectious causes. Cephalosporins, including ceftazidime, have been associated with both immune-mediated hemolytic anemias as well as changes to bone marrow microenvironments both *in vitro* and *in vivo* in mammals (23–25). The turtle in this case received ceftazidime for ~6 weeks prior to the onset of the first anemic event. In order to address the potential for both adverse drug reactions as well as septicemia, ceftazidime was discontinued, a blood culture was obtained, broad-spectrum antimicrobial coverage was initiated with amikacin and ampicillin, and a course of steroids was trialed. The authors have found that some acute anemias in green turtles, as in this case, have been steroid responsive; however, in addition to the immunomodulatory effects of steroids, a possible resolving infection and the cessation of the cephalosporin may have also contributed to improvement. Given that penicillins have also been associated with hemolytic anemia in mammals, it is unlikely, but possible, that this contributed to the second anemic event in this patient (26). There was no clinical evidence of hemorrhage or coagulopathy in either instance. Further investigation into the underlying causes for acute anemias in rehabilitating green turtles is needed in order to aid therapeutic decision making.

## Data Availability Statement

The raw data supporting the conclusions of this article will be made available by the authors, without undue reservation, to any qualified researcher.

## Ethics Statement

Sea turtle rehabilitation was conducted in accordance with Georgia Department of Natural Resources Wildlife Rehabilitation Permit # OS4 CN 1000450736 and Florida Fish and Wildlife Commission's Marine Turtle Permit # MTP-17-135 following general principles of veterinary medicine and animal care.

## Author Contributions

TW contributed to the early clinical case management and completed the first manuscript draft. NIS provided the processing and interpretation for all clinical pathology samples. NLS provided the processing and interpretation for all histological samples. TN managed and oversaw all clinical and surgical aspects of this case. All authors assisted in writing and editing of the manuscript.

### Conflict of Interest

The authors declare that the research was conducted in the absence of any commercial or financial relationships that could be construed as a potential conflict of interest.
